# Isolated Radial Nerve Palsy Following Supracondylar Humerus Fracture in a Pediatric Patient: A Case Report

**DOI:** 10.7759/cureus.72677

**Published:** 2024-10-30

**Authors:** Mohammed Albahlal, Omer Zahid, Abrar Abutaher, Bayan Alsharkhat, Njood Alsudairy

**Affiliations:** 1 General Medicine, Majmaah University, Al Majma'ah, SAU; 2 General Medicine, Royal Bahrain Hospital, Manama, BHR; 3 General Medicine, Salmaniya Medical Complex, Manama, BHR; 4 General Medicine, Southeast University, Jiangsu, CHN; 5 Radiology, The Second Jeddah Health Cluster, Jeddah, SAU

**Keywords:** closed reduction and percutaneous pinning, nerve recovery, pediatric, pediatric elbow injury, radial nerve palsy, supracondylar fracture, wrist drop

## Abstract

Supracondylar fractures of the humerus are the most common elbow fractures in children, with a well-documented risk of associated neurovascular complications. While injuries to the median and anterior interosseous nerves are more frequently reported, isolated radial nerve palsy is a relatively rare complication. We report a case of isolated radial nerve palsy following a displaced supracondylar fracture in a pediatric patient, detailing the clinical presentation, management, and outcome. A six-year-old male presented to the emergency department following a fall from a playground, sustaining a displaced supracondylar fracture of the left humerus. On physical examination, the patient exhibited wrist drop and an inability to extend the fingers, consistent with radial nerve palsy. Imaging confirmed a Gartland Type III supracondylar fracture. Closed reduction and percutaneous pinning (CRPP) were performed, with fracture stabilization achieved. Postoperatively, radial nerve palsy persisted but showed gradual improvement over the subsequent weeks. By six weeks, the patient had regained full wrist and finger extension, and radiographs confirmed fracture healing. The patient completed physical therapy and fully recovered with no residual neurological deficits at the 12-week follow-up. This case underscores the importance of timely recognition and management of nerve injuries in pediatric supracondylar fractures. With appropriate treatment, including fracture reduction and comprehensive rehabilitation, children can achieve full neurological and functional recovery from radial nerve palsy.

## Introduction

Supracondylar fractures of the humerus are among the most common pediatric fractures, accounting for 60-70% of all elbow fractures in children. These fractures typically result from a fall onto an outstretched hand, with the distal humerus experiencing significant force, leading to fracture at the thin, metaphyseal region just above the elbow joint [1,2]. Supracondylar fractures are classified into three types based on the Gartland classification, with Type III being the most severe, involving complete displacement of the fracture fragments and often requiring surgical intervention [2,3].

Neurological complications are a recognized risk in supracondylar fractures, with the radial, ulnar, and median nerves all susceptible to injury due to their close proximity to the fracture site. Radial nerve palsy is a relatively uncommon complication, occurring in approximately 5-10% of cases, and typically arises from traction, contusion, or entrapment of the nerve by the displaced fracture fragments [2,3]. Isolated radial nerve palsy presents with characteristic motor deficits, including wrist drop and the inability to extend the fingers, which can significantly impair upper limb function [1-3].

Early diagnosis and appropriate management of nerve injuries in the context of supracondylar fractures are crucial to optimize outcomes. In most cases, nerve function recovers spontaneously with proper fracture management, but close monitoring and rehabilitation are essential to ensure full functional recovery.

## Case presentation

A six-year-old male presented to the emergency department after falling off a playground structure onto his outstretched hand. He immediately experienced severe pain and swelling in his left elbow, with an inability to move his wrist or extend his fingers. His parents reported that the child was otherwise healthy, with no significant medical history, no previous fractures, and no neurological conditions. The patient had no known allergies or regular medications. His immunizations were up-to-date, and there was no family history of neuromuscular disorders.

On physical examination, the child was visibly distressed, holding his left arm close to his body. Inspection of the left upper limb revealed significant swelling around the elbow, with tenderness and bruising noted over the supracondylar region. There was no evidence of an open wound or deformity of the overlying skin. However, his neurovascular examination raised concerns. While the radial pulse was palpable and the hand was well-perfused, the patient exhibited a complete inability to extend his wrist (wrist drop) or fingers, consistent with a motor deficit of the radial nerve. Sensation testing revealed decreased sensation over the dorsum of the hand, particularly in the first web space, further supporting radial nerve involvement. The remainder of his neurovascular examination, including ulnar and median nerve functions, was intact.

**Figure 1:**
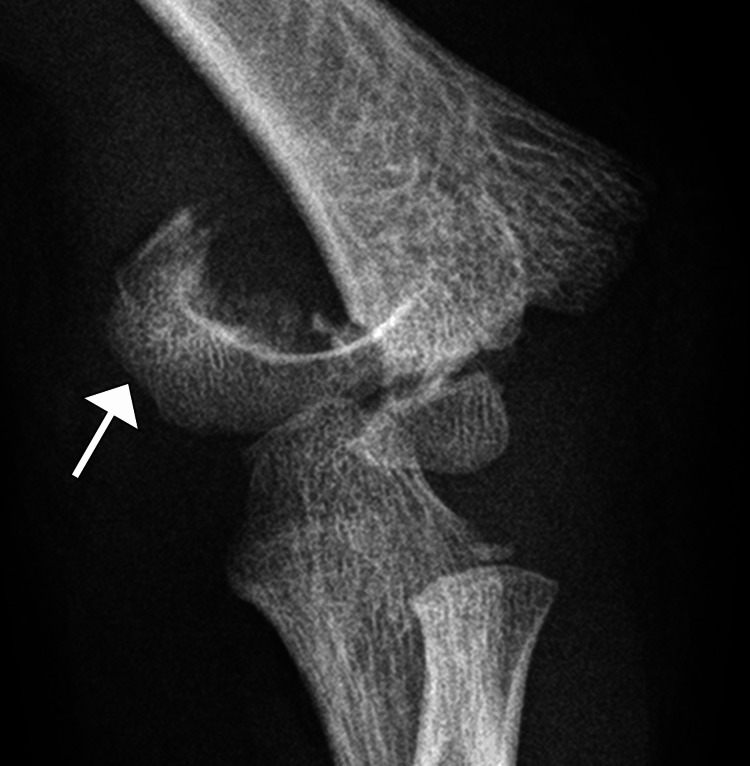
Anteroposterior radiograph of the left elbow Anteroposterior radiograph of the left elbow in a six-year-old patient demonstrating a complete, transversely oriented distal humeral fracture through the olecranon and coronoid fossae (arrow). The image reveals greater than 50% medial translation of the metaphysis, highlighting the significant displacement associated with the fracture.

**Figure 2:**
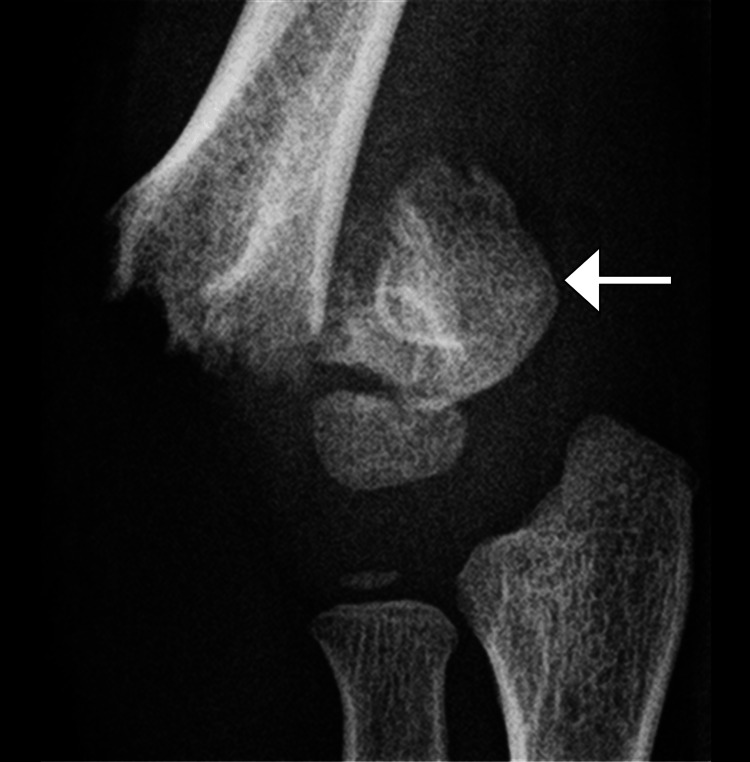
Lateral radiograph of the left elbow Lateral radiograph of the left elbow in a six-year-old patient exhibiting a Gartland Type III supracondylar humeral fracture. The image shows no cortical contact between the posteriorly displaced distal fragment and the proximal fragment (arrow).

Initial radiographs of the elbow were obtained, and a displaced supracondylar fracture of the humerus was demonstrated. It was classified as a Gartland Type III fracture, indicating a completely displaced fracture with no cortical contact. The fracture line was clearly seen extending through the distal humerus, with posterior displacement of the distal fragment. There were no associated fractures of the radius or ulna noted (Figures 1, 2).

Given the clinical presentation of radial nerve palsy in conjunction with the supracondylar fracture, a working differential diagnosis included isolated radial nerve palsy secondary to nerve traction or contusion from the displaced fracture. Other considerations, though less likely, included direct nerve laceration or entrapment within the fracture site. The absence of vascular compromise ruled out complications such as Volkmann’s ischemic contracture.

The patient was taken to the operating room for urgent closed reduction and percutaneous pinning (CRPP) under general anesthesia. Intraoperatively, the fracture was successfully reduced, and two lateral pins were placed to stabilize the fracture. The radial nerve was not directly explored as there was no indication of laceration, and it was presumed that the nerve injury was due to traction or contusion. A long arm cast was applied, immobilizing the elbow at 90 degrees of flexion.

The child was monitored closely in the post-anesthesia care unit. Postoperatively, his radial nerve palsy persisted, with continued wrist drop and inability to extend his fingers. The patient’s pain was controlled with oral analgesics, and he was discharged the next day with an outpatient follow-up scheduled for one week. At follow-up, there was no change in his neurological status.

Over the next several weeks, the patient’s radial nerve function was monitored closely. By the third postoperative week, the patient began to demonstrate gradual improvement in wrist extension, suggesting nerve recovery. By the sixth week, he regained full wrist and finger extension, indicating resolution of the radial nerve palsy. The patient was referred to physical therapy for rehabilitation to restore the full range of motion and strength in the affected arm.

## Discussion

The case of isolated radial nerve palsy following a supracondylar humerus fracture in a pediatric patient presents a relatively rare but important complication that warrants discussion. Supracondylar fractures are the most common type of elbow fractures in children, typically resulting from falls on outstretched hands. While these fractures are frequently encountered, the development of neurological deficits, particularly involving the radial nerve, is less commonly reported [3,4]. This case highlights the importance of early recognition, accurate diagnosis, and timely management of such injuries to optimize neurological recovery and functional outcomes.

Radial nerve injuries in the context of supracondylar fractures are often attributed to traction or contusion of the nerve, particularly in cases of Type III fractures where the displacement of fracture fragments can result in significant soft tissue and nerve stretch. Although radial nerve injuries are less common than those involving the median or anterior interosseous nerve, they can have profound functional consequences due to the nerve’s role in wrist and finger extension [2-4]. Isolated radial nerve palsy, as seen in this patient, presents with classic signs such as wrist drop and sensory deficits in the dorsum of the hand. The pathophysiology of these injuries typically involves nerve traction rather than direct laceration, as confirmed by this patient’s ultrasound findings, which demonstrated no nerve discontinuity or severe compression [2,5].

The management of supracondylar fractures with concomitant nerve injuries remains a subject of debate. Traditionally, the majority of nerve palsies associated with these fractures are managed conservatively, with the expectation of spontaneous recovery as fracture alignment is restored. Studies have demonstrated that up to 90% of nerve injuries associated with supracondylar fractures recover within three to six months post-injury without the need for surgical exploration. In the present case, the decision to proceed with CRPP was guided by the fracture classification (Gartland Type III) and the lack of vascular compromise, rather than the presence of radial nerve palsy [3-6]. Consistent with existing literature, the nerve injury resolved over time, with the patient regaining full wrist and finger extension by six weeks postoperatively, a timeline in line with documented cases of radial nerve recovery.

Nevertheless, there are circumstances where surgical exploration of the nerve may be warranted, particularly if there is no clinical or electrophysiological evidence of recovery within three months, or if there are signs of nerve entrapment by the fracture or scar tissue [2-5]. In this case, the decision not to explore the nerve intraoperatively was supported by the absence of vascular injury and the clinical presentation of a traction injury, which carries a favorable prognosis for spontaneous recovery.

This case also emphasizes the importance of a multidisciplinary approach to care, including postoperative rehabilitation. Physical therapy played a crucial role in this patient's recovery, helping restore the full range of motion and strength in the affected limb following the resolution of the nerve palsy. This highlights the broader principle that optimal outcomes in pediatric fractures with nerve injuries are achieved through not only timely surgical intervention but also comprehensive postoperative care, including neurorehabilitation.

From a broader perspective, the literature supports the notion that early and effective fracture stabilization through CRPP is the standard of care for displaced supracondylar fractures. This technique is associated with excellent outcomes, minimal complications, and a low risk of iatrogenic nerve injury. The present case reinforces the efficacy of this approach in managing pediatric supracondylar fractures complicated by nerve palsy, further supporting its role as the treatment of choice in such scenarios.

## Conclusions

In conclusion, this case illustrates the importance of early recognition and appropriate management of isolated radial nerve palsy in pediatric supracondylar fractures. Given the excellent prognosis of traction-related nerve injuries, a conservative approach focusing on fracture reduction and close neurological monitoring is appropriate in most cases. Surgical exploration should be reserved for cases with delayed nerve recovery or signs of nerve entrapment. With a high index of suspicion, prompt treatment, and comprehensive rehabilitation, children with this rare complication can expect full functional recovery, as demonstrated in this case. Further research is needed to refine the indications for nerve exploration and optimize rehabilitation strategies for nerve injuries associated with pediatric fractures.
